# Can we stop malaria parasites in the skin?

**DOI:** 10.1186/1475-2875-13-S1-O7

**Published:** 2014-09-22

**Authors:** Ross Douglas, Miriam Ester, Janina Hellmann, Friedrich Frischknecht

**Affiliations:** 1 Centre for Infectious Diseases, Heidelberg University, Heidelberg 69120, Germany

## Background

Since the discovery of malaria transmission by mosquitoes, it was assumed that the parasites are injected directly into the blood stream. However, indirect experiments [1] and direct microscopic observations using mice as hosts and fluorescent rodent malaria species showed that the parasites are instead injected into the skin. These *Plasmodium* sporozoites then migrate rapidly through the dermis and enter blood or lymph vessels [2]. Stopping sporozoite motility also halts infection [3]. We aim at understanding the mechanisms that drive sporozoite motility and identify drug-like compounds that stop parasite locomotion. To this end, we have adapted and developed new methods including a screening pipeline to test small molecules that could interfere with motility and thus stop *Plasmodium* transmission at the skin stage [4,5].

## Materials and methods

A screening pipeline was developed that allowed medium-throughput assessment of small molecules as possible inhibitors of sporozoite motility *in vitro*. This was followed by *in vivo* testing during transmission from mosquito to mouse.

## Results

We tested over 200 compounds selected from a library of drugs approved by the Federal Drug Administration for their potential to interfere with motility. We identified two molecules that inhibited *in vitro* motility in the nano-molar range. When these two compounds were tested during the transmission by mosquitoes, an ectopically applied drug resulted in a decrease of transmission efficiency while an orally given drug showed no effect on transmission at non-toxic doses.
